# Parental determinants of blastocyst euploidy in a predominantly recurrent-pregnancy-loss PGT-A cohort: limited predictive value of female age, BMI, AMH and sperm DNA fragmentation

**DOI:** 10.3389/fendo.2026.1909930

**Published:** 2026-10-28

**Authors:** Chang Tan, Xiliang Wang, Dan Tong, Pengshu Zou, Xun Liu, Yuexin Yu

**Affiliations:** 1 Department of Reproductive Medicine, General Hospital of Northern Theater Command, Shenyang, China; 2 Department of General Surgery, Shengjing Hospital of China Medical University, Shenyang, China

**Keywords:** embryo euploidy, female age, generalized linear mixed model, PGT-A, predictive model, sperm DNA fragmentation index

## Abstract

**Background:**

Embryonic aneuploidy is a major factor affecting clinical outcomes in assisted reproduction. PGT-A improves embryo selection, but the clinical factors associated with euploidy remain debated.

**Methods:**

This retrospective study included 588 PGT‑A cycles. We used embryo‑level GLMMs to evaluate the associations of female age, female BMI, AMH, male age, male BMI, and sperm DFI with embryo euploidy. We performed univariable and multivariable analyses, assessed model performance (AUC, R^2^, calibration), and conducted internal validation via patient-clustered bootstrap (200 replicates). Subgroup analyses by female age were performed with FDR correction.

**Results:**

Among 2,357 blastocysts, 1,299 (55.1%) were euploid. In univariable analysis, female age, male age, and male DFI were significantly associated with euploidy. After multivariable adjustment, only female age (OR = 0.87, p = 0.011) and male DFI (OR = 0.89, p = 0.006) remained independent predictors. Model performance was poor (apparent AUC = 0.571; bootstrap-corrected AUC = 0.574; apparent marginal R^2^ = 0.018 and conditional R^2^ = 0.036; apparent calibration slope = 0.985; bootstrap-corrected calibration slope = 0.913), indicating weak signal strength and limited generalizability. Subgroup analyses showed some nominal associations, but none survived FDR correction (all q > 0.05). Among 588 cycles with complete covariate data, 90.3% had at least one euploid blastocyst; among all 613 biopsied cycles and 637 started cycles, the rates were 89.89% and 86.50%, respectively.

**Conclusion:**

Female age and male DFI were independently associated with euploidy, but the model’s discrimination remained poor after optimism correction (bootstrap-corrected AUC = 0.574; apparent calibration slope = 0.985, bootstrap-corrected calibration slope = 0.913), and these variables therefore lack sufficient value for clinical use. Subgroup analyses by female age were exploratory and hypothesis-generating, and no statistically significant age-modified association between male DFI and euploid embryos persisted after FDR correction. Future models incorporating embryo-specific data and larger samples are needed.

## Introduction

An increasing number of couples with infertility have successfully achieved pregnancy through assisted reproductive technology (ART). However, early embryonic arrest and recurrent implantation failure remain significant challenges in assisted reproduction. Among the various contributing factors, embryonic aneuploidy is the primary cause of implantation failure and miscarriage (1–3). PGT-A enables the prioritization of euploid embryos for transfer, thereby improving clinical pregnancy outcomes, particularly in women of advanced maternal age, those with unexplained recurrent pregnancy loss, and those who experience repeated unsuccessful implantation. A higher euploidy rate indicates that more embryos are available for transfer, with a correspondingly higher cumulative pregnancy success rate. Therefore, identifying the relevant factors associated with euploid embryos remains the key focus in reproductive medicine.

Previous studies have reported multiple factors associated with the euploidy rate. Female age is widely recognized as the most important risk factor (4–6), and the underlying mechanism involves chromosomal segregation errors during oocyte meiosis. Whether anti-Müllerian hormone (AMH), a marker of ovarian reserve, directly reflects oocyte quality remains controversial (7–9). The relationship between female body mass index (BMI) and embryo euploidy is inconclusive (10–13): Some studies suggest that obesity may induce oxidative stress in the follicular microenvironment and increase the risk of aneuploidy, while others have found no independent association. The effects of male age and sperm DNA fragmentation index (DFI) on embryo chromosomal status are still debated (14–17), however the independent role of male age is often masked by female age. In addition, studies evaluating the association between male BMI and euploidy rate remain scarce.

Furthermore, current studies on euploidy predominantly focus on one or two isolated factors, lacking a comprehensive multivariable comparison. To date, few studies have simultaneously investigated the associations between blastocyst euploidy rate and six variables: female age, female BMI, AMH, male age, male BMI, and male DFI. Therefore, this study focused exclusively on these six pre-treatment baseline characteristics, some of which are modifiable. To avoid over-adjustment bias when estimating the independent effects of pre-treatment parental baseline factors, our primary model did not include ART procedural parameters (e.g., ovarian-stimulation protocols, total oocytes retrieved) or downstream embryological variables (e.g., blastulation day and blastocyst morphological grade). However, to assess the robustness of our findings to potential confounding by these factors, we performed exploratory sensitivity analyses incorporating blastulation day, morphological grade, and infertility type.

## Materials and methods

### Study design and population

This study included patients who underwent PGT-A treatment at our department between January 2020 and December 2023. Inclusion criteria were: (1) only the first PGT-A cycle was included for each enrolled patient, (2) at least one blastocyst that successfully underwent biopsy and genetic testing. Based on expert consensus, the clinical indications for PGT-A in assisted reproductive treatment are as follows: advanced maternal age (defined by our department as ≥ 37 years for PGT-A referral criteria), recurrent pregnancy losses (RPL), or unexplained recurrent implantation failure (RIF). RPL was defined as ≥ 2 consecutive clinical pregnancy losses, and RIF as ≥ 3 failed embryo transfers with high-quality embryos (18, 19).

The exclusion criteria were as follows: (1) blastocysts identified as mosaic embryos through PGT-A testing; (2) partial PGT testing of blastocysts because of patient preference; (3) no blastocysts were suitable for biopsy, and (4) missing clinical data. A total of 588 patients (588 PGT-A cycles) were ultimately included, with each patient contributing only one cycle. The analytical unit of this study was the embryo level, with a total of 2357 blastocysts from the above 588 cycles included.

All patients underwent genetic counselling. This study is a retrospective study, which was conducted in accordance with the Declaration of Helsinki. This retrospective study analyzed clinical data at our department between January 2020 and December 2023. The study protocol was approved by the Medical Ethics Committee of the General Hospital of the Northern Theater Command [Y (2026) 84] for the retrospective analysis of de-identified data; all research activities were initiated after approval. The requirement for informed consent was waived by the ethics committee because this study involved only the analysis of de-identified clinical data generated from past routine practice, with no additional intervention or contact with the participants.

Given the retrospective design and fixed nature of this cohort, the statistical adequacy of the model was evaluated using the events-per-variable (EPV) criterion. The primary multivariable GLMM included 6 predictors and was based on 1,299 euploid blastocyst events, yielding an EPV of approximately 216 (1,299/6), which substantially exceeds the recommended minimum of 10–15 events per predictor, indicating sufficient power for estimating the main effects.

Age was stratified as < 35, 35-39, and ≥ 40 years (distinct from the clinical AMA threshold of ≥ 37 years) to assess the biological gradient. The euploid event counts across the three strata were 595, 566, and 138, respectively, yielding stratum-specific EPV values ranging from approximately 23 to 99. Although these EPV values still meet the nominal minimum requirement, the power to detect modest interaction or subgroup effects is limited due to the reduced sample size per stratum and the larger number of multiple comparisons (18 tests). All subgroup p-values were adjusted using the Benjamini-Hochberg method, and adjusted q-values are reported. Therefore, all subgroup analyses were pre-specified as exploratory and hypothesis-generating, and the corresponding results should be interpreted with caution.

### Sperm DFI testing

Semen samples were collected on the day of oocyte retrieval after an ejaculatory abstinence period of 2–7 days, according to the WHO Laboratory Manual for the Examination and Processing of Human Semen (6th edition) (20). After liquefaction at room temperature for 30–60 minutes, one aliquot was used for routine semen analysis and ICSI procedures and another aliquot was used for DFI testing. Sperm DNA fragmentation index (DFI) was evaluated using acridine-orange flow-cytometric chromatin structure assessment. Briefly, after dilution and acid-induced DNA denaturation, sperm samples were stained with acridine orange using a commercially available kit (Sperm Nuclear Integrity Staining Kit, Zhejiang Xingbo Biotechnology Co., Ltd., China) and analyzed on a BD Accuri C6 flow cytometer (Becton Dickinson). The DNA fragmentation index (DFI) was calculated based on the metachromatic shift of acridine orange fluorescence from green (native double-stranded DNA) to red (denatured single-stranded DNA), using the formula: DFI (%) = red fluorescence/(green fluorescence + red fluorescence) × 100.

The DFI assay was completed within 2 hours of ejaculation. Since oocyte retrieval was performed on the same day, the time interval between DFI testing and oocyte retrieval was consistently less than 4 hours for all patients. Only a single DFI measurement was performed per patient using the ICSI-day sample. Strict internal quality control (QC) procedures were implemented throughout the study. Fixed positive and negative control slides were run with each batch of patient samples to monitor assay performance.

### Ovarian stimulation and embryo culture

All patients underwent ovarian induction using an appropriate protocol. Transvaginal ultrasound-guided oocyte retrieval was performed 36 h after the human chorionic gonadotropin (hCG) administration. Intracytoplasmic sperm injection was used for fertilization, and fertilization status was assessed on day 1 post-retrieval. The presence of two pronuclei indicated normal fertilization. The injected oocytes were transferred to individual microdroplets of cleavage medium (Quinn’s Advantage Protein Plus Cleavage Medium; CooperSurgical) and cultured under oil at 37 °C in an atmosphere containing 5% O_2_ and 6% CO_2_. On day 3, the culture medium was replaced with blastocyst medium (Quinn’s Advantage Blastocyst Medium; CooperSurgical) for sequential culture. Biopsy sampling of blastocyst-stage embryos (trophectoderm) was performed on day 5, 6, or 7 after fertilization, among all 2,357 biopsied blastocysts, 96% were biopsied on day 5 or day 6 and 4% on day 7. Following biopsy, embryos were individually cryopreserved via vitrification (Kitazato vitrification kit).

### Blastocyst biopsy

Two senior embryologists scored blastocyst morphology based on the Gardner scoring system (21). Throughout the study period (January 2020 - December 2023), the criterion for performing trophectoderm biopsy was defined component-wise and did not change: a blastocyst was biopsied when its expansion grade was at least 4 and at least one of the two lineages-inner cell mass or trophectoderm-was graded A or B. Blastocysts graded CC in both lineages were not biopsied and therefore did not enter any analysis dataset. Consistent with this rule, low quality in the analyses refers to blastocysts with exactly one lineage graded C. High-quality blastocysts were defined as those in which neither the trophectoderm (TE) nor the inner cell mass (ICM) was graded as C (22). A ZILOS-tk Hamilton Thorne laser system (Hamilton Thorne, Inc., Beverly, MA, USA) was used to create an opening in the zona pellucida, followed by the extraction and separation of 3–5 TE cells using a biopsy needle. TE cells were placed in a 0.2-mL PCR tube containing 2 μL phosphate-buffered saline (Thermo Fisher Scientific, Waltham, MA, USA) and stored at -20 °C. Biopsied embryos were screened for euploidy using a targeted PGT-A platform based on next-generation sequencing (NGS) technology.

### NGS sequencing

NGS was performed to analyze TE biopsy samples. Single-cell whole-genome amplification (WGA) was performed using the SurePlex (Takara Bio, Inc., Tokyo, Japan). The WGA products underwent library preparation, including fragmentation, adapter ligation, PCR amplification, and purification. Purified libraries were quantified using Qubit (Thermo Fisher Scientific, Waltham, MA, USA) and sequenced on an Illumina MiSeq DX platform (Illumina, San Diego, CA, USA). The PGX Cloud data analysis system (Illumina) was used for aneuploidy assessment. Based on sequencing results, blastocysts were classified as euploid, aneuploid, or mosaic. Embryo classification was performed using the 20-80 % mosaic-range criteria (20-80 % aneuploid cell fraction in TE biopsy). A blastocyst was reported as mosaic only when the following four conditions were simultaneously met: (i) an aneuploid cell fraction within the 20–80% range; (ii) successful whole-genome amplification; (iii) sequencing quality metrics passing laboratory thresholds (MAPD < 0.30 with adequate on-target coverage); and (iv) concordant independent review by two analysts. Intermediate-copy-number deviations attributed by dual review to technical artefacts (amplification bias, regional coverage unevenness) were resolved to euploid or aneuploid rather than reported as mosaic. In the 588 complete-data cycles, all 2,369 biopsied blastocysts received one of the three definitive calls (12 mosaic, 1,299 euploid, 1,058 aneuploid) from the 588 complete-data cycles; no sample remained unclassified. The 71 blastocysts from the 25 excluded cycles likewise all received a definitive call (40 euploid, 31 aneuploid, 0 mosaic). In this study, only euploid and completely aneuploid blastocysts were included, while mosaic blastocysts were excluded.

### Statistical analysis

To account for the hierarchical data structure (multiple embryos per patient), we used generalized linear mixed models (GLMMs) with a binomial distribution and logit link. The dependent variable was embryo euploidy status (1 = euploid, 0 = aneuploid). Patient identity was included as a random intercept to adjust for within‑patient correlation. All continuous predictors were standardized (mean = 0, SD = 1) to improve convergence and effect size comparability.

Univariable GLMMs were fitted for each predictor, followed by a multivariable model including all six predictors simultaneously. All models were estimated using the glmer function (lme4 package, bobyqa optimizer, 200,000 iterations). Results are reported as odds ratios (ORs) with 95% CIs and two‑sided p‑values; the OR reflects the effect per 1-SD increase.

Model performance was evaluated using AUC, marginal/conditional R^2^, and calibration plots. The AUC values reported in this study are based on marginal predictions (with random effects set to zero) and are consistent across both the apparent sample and the bootstrap samples. It is also confirmed that the model was fully refitted in each bootstrap iteration. Calibration was quantified by calibration intercept (ideal: 0) and calibration slope (ideal: 1), and overall accuracy by the Brier score. To correct for optimism, internal validation was performed using patient-ID-clustered bootstrap resampling (200 iterations): in each replicate, patients were resampled with replacement at the patient level, the model was fully refitted on the bootstrap sample, and predictions for the original cohort were evaluated on the marginal scale (random effects set to zero), so that the optimism-corrected metrics are directly comparable with the apparent values. All analyses were conducted in R version 4.3.2 using the lme4, pROC, and performance packages.

Subgroup analyses were performed by stratifying female age into three groups: < 35, 35-39, and ≥ 40 years. For each stratum, the multivariable GLMM was refitted. Interaction analyses tested female‑age × male-DFI and female-BMI × male-BMI in separate models. To complement the embryo-level analysis, we also calculated the proportion of cycles yielding at least one euploid blastocyst (per-cycle outcome) using simple proportions (**Table 1**).

**Table 1:** Demographic characteristics of the patients who underwent PGT-A cycles.

Characteristics	Description
Number of PGT-A cycles (n)	588
Number of biopsied blastocysts	2357
Euploidy blastocysts	1299
Euploidy rate (%)	55.11%
Female age (years)	35.76 ± 3.95
Female BMI (kg/m^2^)	23.68 ± 3.53
Female AMH (ng/mL)	3.23 ± 2.91
Type of infertility, n (%)	
Primary	157(26.70%)
Secondary	431(73.30%)*
Male age (years)	36.90 ± 5.26
Male DFI (%)	12.63 ± 5.81
Male BMI (kg/m^2^)	25.82 ± 3.80
Cycles with ≥1 euploid blastocyst among 588 complete-data cycles	531/588 (90.31%)
Cycles with ≥1 euploid blastocyst among 613 biopsied cycles	551/613 (89.89%)
Cycles with ≥1 euploid blastocyst among 637 started cycles	551/637 (86.50%)
Indication for PGT-A,	
n (%)	80(13.61%)
Advanced maternal	416(70.75%)
Recurrent pregnancy loss	92(15.64%)
Repeated implantation failure	431(73.30%)*
Low DFI (<15%)	151(25.68%)
Intermediate DFI (15%– 30%)	6(1.02%)
High DFI(≥30%)	2.21
Mean number of euploid embryos per cycle	9.69%

PGT-A, preimplantation genetic testing for aneuploidies; BMI, body mass index; AMH, anti-Müllerian hormone; DFI, sperm DNA fragmentation index. Values are expressed as mean ± SD or n (percentage%).

*The identical counts and percentages for secondary infertility and DFI <15% are derived from independent statistical analyses. Double-verification of the original data confirmed no transcription errors; the two subgroups are not fully overlapping.

90.31% refers only to the 588 cycles with complete covariate data. The 25 cycles excluded for missing covariates had PGT-A results; 20 had ≥1 euploid blastocyst. Therefore, among all 613 biopsied cycles and all 637 started cycles, 551 (89.89%) and 551 (86.50%) had ≥1 euploid blastocyst, respectively.

To assess collinearity among fixed predictors, we calculated variance inflation factors (VIFs), with thresholds >5 indicating potential issues and >10 indicating severe multicollinearity. Since GLMMs do not directly provide VIFs, we fitted a standard logistic regression (without random intercept) to embryo-level data and computed VIFs using the vif function from the R package car. This approach is valid because VIFs depend only on the fixed-effect design matrix. Results are presented in **Supplementary Table S6**.

All analyses were conducted using R software, version 4.3.2, with the packages lme4 (v1.1‑34), broom.mixed (v0.2.9.4), pROC (v1.18.5), performance (v0.10.8), ggplot2 (v3.4.4), and patchwork (v1.1.3).

### Sensitivity analyses

To evaluate the confounding effects of embryo-level characteristics, we performed exploratory sequential sensitivity analyses using GLMMs with patient random intercepts. All continuous predictors were standardized. Model 2 added blastulation day (D5 [reference], D6, D7). Model 3 further added morphological grade (high quality [ICM and TE both graded A or B] vs. low quality [either ICM or TE graded C]), and Model 4 additionally included infertility type(secondary vs. primary, with primary as the reference). PGT-A indication was deliberately not included as a covariate, because one of its three categories — advanced maternal age (AMA), defined in our center as ≥ 37 years — is a deterministic function of female age; adjusting for it would absorb the age effect by construction and render female age artificially non-significant. Due to incomplete historical records, morphological data were available for 1,819 of the original 2,357 blastocysts (77.2%); thus, all four models were based on that subset (**Supplementary Table S7**).

To distinguish selection from adjustment, the primary six-predictor model (Model 1) was refitted on the same 1,819 - embryo subset and compared with the full-cohort estimate (**Supplementary Table S9**); in addition, Models 1 and 2 were refitted on all 2,357 embryos, for which blastulation day was recorded (**Supplementary Table S9**). **Supplementary Table S7** is therefore reported as an exploratory, secondary analysis.

## Results

### Study selection

A total of 637 PGT-A cycles were initially screened between January 2020 and December 2023. As shown in **Figure 1**, 24 cycles formed no blastocyst, leaving 613 biopsied cycles. Of these, 25 cycles were excluded from the primary analysis because of missing BMI, AMH, or DFI, leaving 588 cycles with complete covariate data. PGT-A results were available for the 25 excluded cycles, which contributed a further 71 biopsied blastocysts (40 euploid, 31 aneuploid, 0 mosaic); 20 of these 25 cycles had at least one euploid blastocyst. These embryos are not included in any analysis dataset because the maternal or paternal covariates required for modelling were missing. 551 of 613 biopsied cycles, 551 of 637 started cycles, and 531 of 588 complete-data cycles had at least one euploid blastocyst. After excluding the 12 mosaic blastocysts identified among the complete-data cycles, 2,357 blastocysts from those 588 cycles were included in the final analysis. In total, 2,440 blastocysts were biopsied across all 613 biopsy cycles.

**Figure 1:**
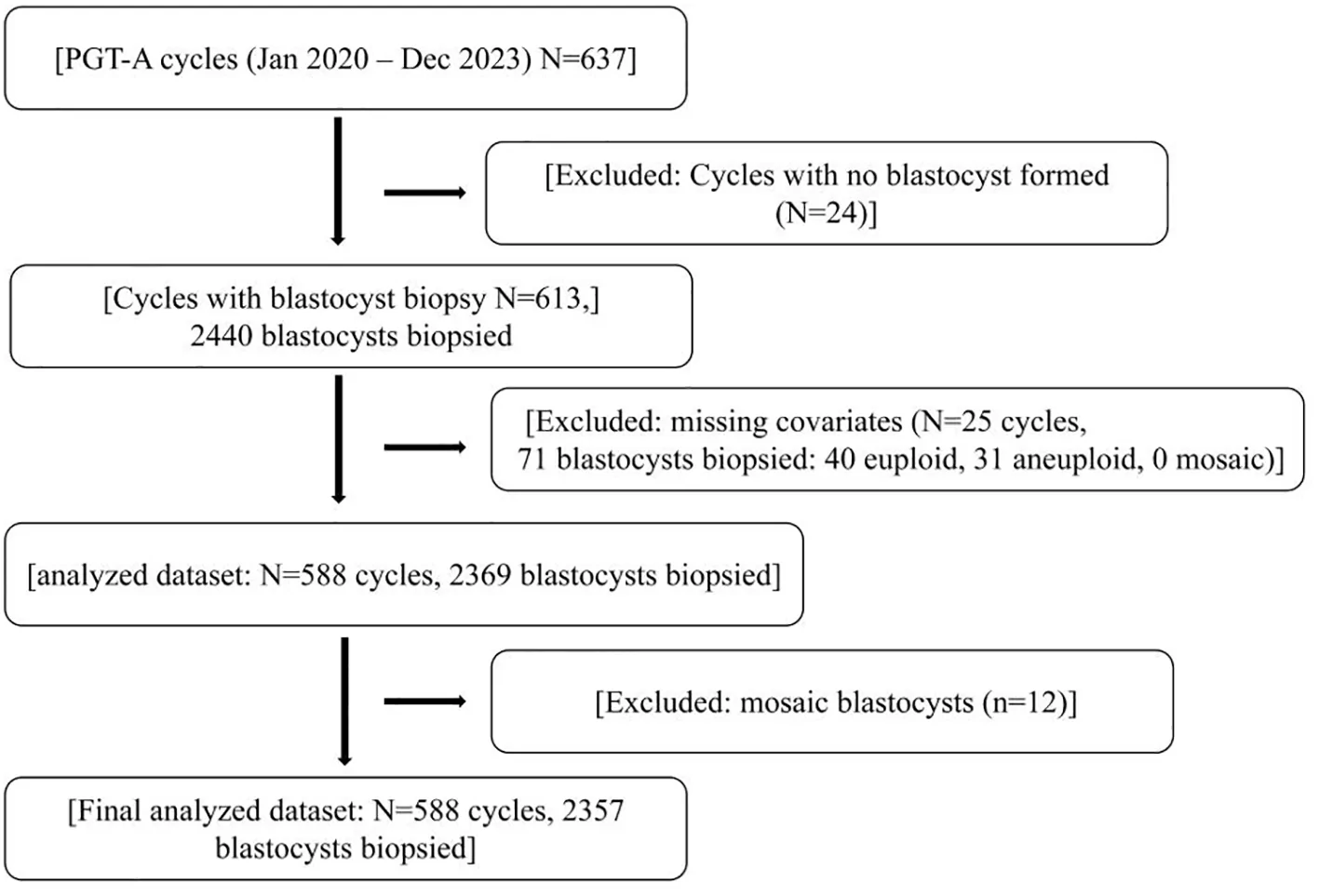
Flowchart of study inclusion and exclusion. Flowchart of cycle and blastocyst inclusion and exclusion. Of 637 PGT-A cycles started, 24 formed no blastocyst, leaving 613 cycles in which blastocysts were biopsied (2,440 blastocysts in total: 1,339 euploid, 1,089 aneuploid, 12 mosaic). Twenty-five cycles were excluded from the primary analysis because of missing BMI, AMH or DFI; their PGT-A results were complete and contributed 71 biopsied blastocysts (40 euploid, 31 aneuploid, 0 mosaic), shown in the grey panel. The primary analysis therefore comprises 588 cycles and 2,369 biopsied blastocysts; after exclusion of 12 mosaic embryos, 2,357 blastocysts were analyzed.

### Demographic characteristics of the patients who underwent PGT-A cycles

A total of 588 cycles with 2,357 blastocysts were included in this study, among these, 1,299 were classified as euploid blastocysts; the overall euploidy rate was 55.1% (1,299/2,357). The cohort was RPL‑predominant (70.75%), with AMA (13.61%) and RIF (15.64%). The mean age of female partners was 35.76 ± 3.95 years, so age-related findings are interpreted specifically within this RPL-dominant, moderately-aged population. Among the 588 cycles with complete covariate data, 531 (90.31%) had at least one euploid blastocyst. When all 613 biopsied cycles and all 637 started cycles were considered, the corresponding rates were 551/613 (89.89%) and 551/637 (86.50%), respectively. The mean number of euploid embryos per cycle is 2.21 (**Table 1**).

### Embryo‑level GLMM analysis

A total of 2,357 blastocysts from 588 cycles were analyzed. In univariable GLMMs, female age (OR = 0.82 [0.76-0.90], p < 0.001), male age (OR = 0.83 [0.77-0.91], p < 0.001), and male DFI (OR = 0.86 [0.79-0.94], p < 0.001) were significantly associated with lower odds of euploidy (**Figure 2A**). Female BMI, male BMI, and female AMH were not significantly associated with euploidy. ORs were for a 1-SD increase in the predictor.

**Figure 2:**
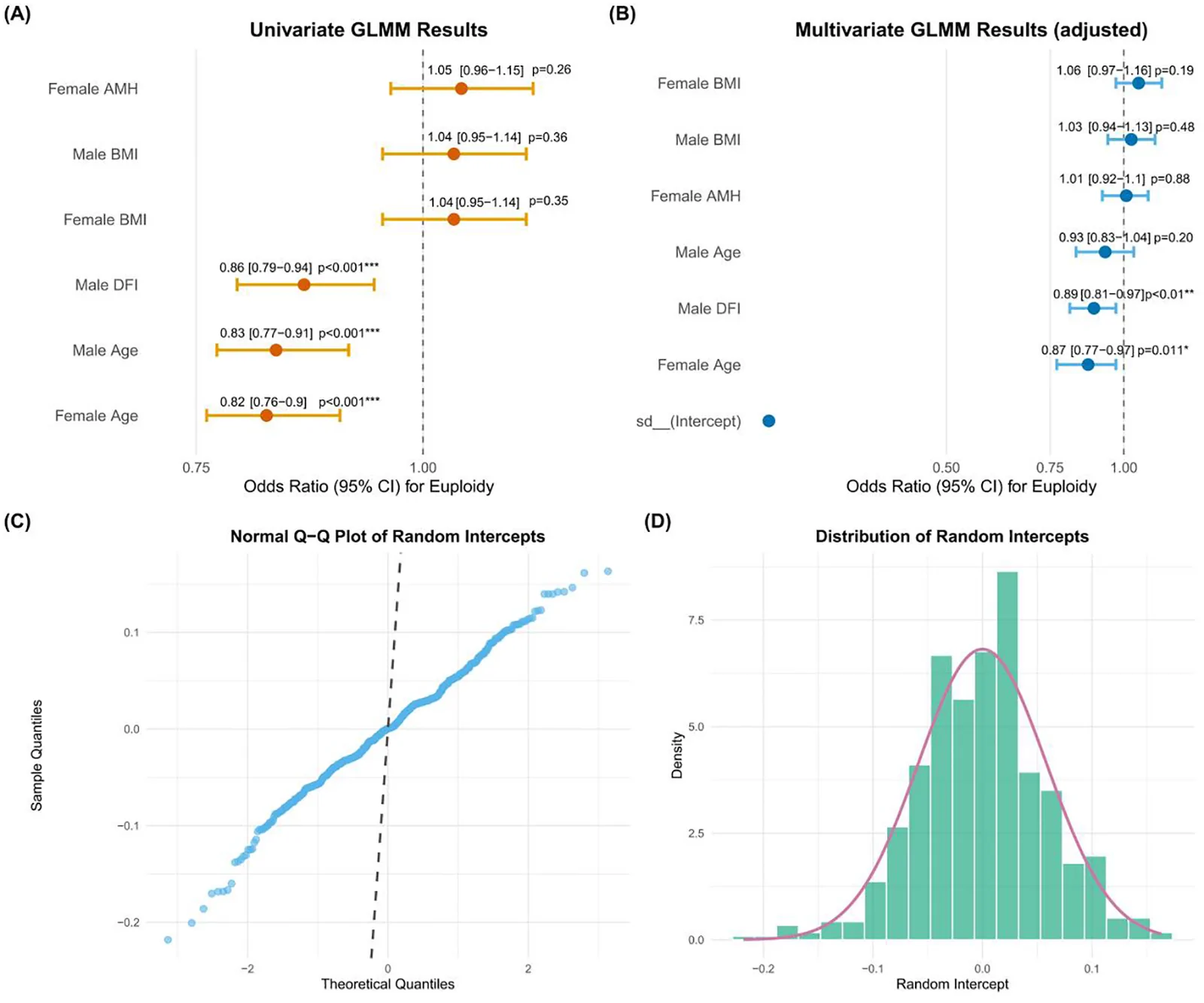
Embryo-level generalized linear mixed-effects model (GLMM) results for factors associated with euploidy. **(A)** Univariable forest plot of odds ratios (ORs) with 95% confidence intervals (CIs) for each predictor, estimated using separate GLMMs with patient random intercepts. ORs <1 indicate lower odds of euploidy. Significance levels: ***p < 0.001, **p < 0.01, *p < 0.05. **(B)** Multivariable forest plot showing adjusted ORs from a single GLMM including all six predictors simultaneously. **(C)** Normal quantile-quantile (Q-Q) plot of the patient random intercepts from the multivariable model; points largely follow the diagonal line, supporting the normality assumption. **(D)** Histogram of random intercepts with overlaid normal density curve. The spread of the shrunken empirical-Bayes estimates is small, consistent with the modest random-intercept SD (0.25; ICC ≈ 0.02). All continuous predictors were standardized; ORs represent the change in odds per 1 standard deviation increase.

In the multivariable GLMM, when the standardized ORs were back‑transformed to clinically intuitive units, female age (per 1-year increase) yielded an adjusted OR of 0.965 (95% CI: 0.936-0.992, p = 0.011), and male DFI (per 5% absolute increase) yielded an adjusted OR of 0.905 (95% CI: 0.834-0.974, p = 0.006) (**Table 2**).

**Table 2:** Association of clinical variables with embryo euploidy status using embryo-level GLMMs with patient random intercepts.

	GLMM with patient random intercept		GLMM with patient random intercept	
Predictor	Univariate.OR.95%CI.	Univariate.p	Multivariate.OR.95%CI.	Multivariate.p
Female Age	0.82 [0.76-0.9]***	<0.001	0.87 [0.77-0.97]*	0.011
Female BMI	1.04 [0.95-1.14]	0.352	1.06 [0.97-1.16]	0.188
Male Age	0.83 [0.77-0.91]***	<0.001	0.93 [0.83-1.04]	0.204
Male BMI	1.04 [0.95-1.14]	0.364	1.03 [0.94-1.13]	0.478
Female AMH	1.05 [0.96-1.15]	0.262	1.01 [0.92-1.1]	0.884
Male DFI	0.86 [0.79-0.94]***	<0.001	0.89 [0.81-0.97]**	0.006

***p<0.001; **p<0.01; *p<0.05. GLMMS, generalized linear mixed models. All predictors were standardized (mean=0, SD = 1) prior to analysis. The random intercept standard deviation was 0.25(ICC ≈ 0.02), indicating small between-patient heterogeneity.

Data were standardized to 1-SD before analysis. Raw SDs used for standardization: Female Age = 3.95 years; Male DFI = 5.81%. To aid clinical interpretation, the multivariable-adjusted ORs per 1-SD were converted: e.g., Female Age per 1-year increase: 0.965 [0.936-0.992]; Male DFI per 5% increase: 0.905 [0.834-0.974].

Variance inflation factors for all fixed predictors were well below the conventional threshold of 5 (range: 1.04-1.69; **Supplementary Table S6**), indicating no substantial collinearity among them, and also ensuring that the estimated coefficients are not inflated by redundant correlations between the covariates. In the multivariable GLMM simultaneously adjusting for all six predictors (**Table 2**), only female age (OR = 0.87 [0.77-0.97], p = 0.011) and male DFI (OR = 0.89 [0.81-0.97], p = 0.006), remained statistically significant. Other factors (male age, female AMH, female BMI, male BMI) were not statistically significant. These results are illustrated in **Figure 2B** (forest plots).

Visual inspection of the Q-Q plot (**Figure 2C**) indicated no significant deviation from normality. This was further supported by the histogram of the random effects (**Figure 2D**), together providing supportive evidence for the appropriateness of the random intercept structure in the GLMM.

### Model performance

**Figure 3** presents the discrimination and calibration performance of the model for predicting embryo euploidy probability. Overall model performance was very low: apparent marginal R^2^ = 0.018, apparent conditional R^2^ = 0.036, and apparent AUC = 0.571(**Supplementary Table S1**). On the marginal prediction scale, apparent calibration was good (apparent intercept −0.005; apparent calibration slope 0.985; **Supplementary Table S4**). After bootstrap correction the calibration slope was 0.913, indicating that the apparent coefficients are approximately 9.5% too extreme on the log-odds scale. Discrimination on the marginal prediction scale was likewise poor. These metrics indicate that the included variables explain only a minimal proportion of the variability in embryo euploidy (**Figure 3**).

**Figure 3:**
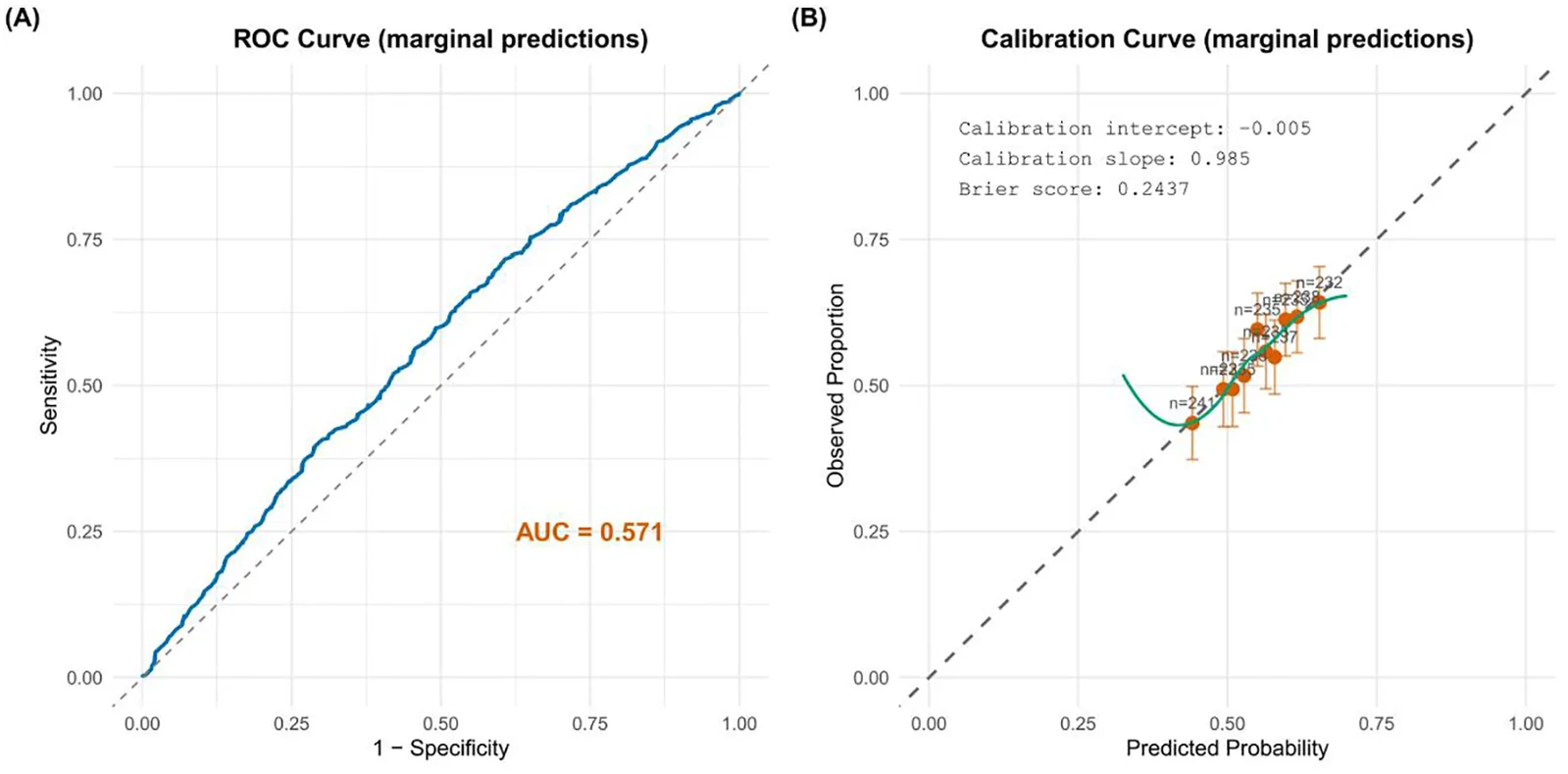
Model performance for predicting blastocyst euploidy. **(A)** ROC curve for the multivariable GLMM predicting embryo euploidy, based on marginal predictions (fixed effects only; patient random intercept set to zero). The apparent AUC is 0.571. **(B)** Calibration curve for the same model, also based on marginal predictions. Points represent deciles of predicted probability; error bars indicate 95% confidence intervals; numbers above points indicate the number of embryos per decile. The dashed diagonal line represents perfect calibration; the green line is a loess smoother. Apparent calibration intercept (−0.005), apparent calibration slope (0.985), and apparent Brier score (0.2437) are displayed in the upper-left corner. Apparent calibration was good, whereas the bootstrap-corrected slope was 0.913 (**Supplementary Table S4**). The model shows good calibration but poor discrimination (AUC ≈ 0.57), consistent with the low marginal R2. Both panels are derived from the primary dataset of 2,357 embryos (mosaic embryos excluded), using marginal predictions.

### Internal validation

Internal validation (200-replicate patient-clustered bootstrap) revealed the following: the apparent AUC was 0.571 and the bootstrap-corrected AUC was 0.574, the observed optimism (−0.003) lies within Monte Carlo error (≈0.002-0.004), i.e. no detectable overfitting. The Brier score was 0.2437 (apparent) and 0.2433 (bootstrap-corrected), marginal explanatory power was low (R^2^ = 0.018). The apparent calibration slope was 0.985 and the bootstrap-corrected slope 0.913 (slope optimism 0.072; **Supplementary Table S4**), indicating mild optimism rather than gross miscalibration. These findings were corroborated by the calibration curve (**Figure 3B**; **Supplementary Table S4**), suggesting limited model performance and clinical utility.

To enhance clinical interpretability, we additionally calculated odds ratios for clinically meaningful increments of continuous predictors. The resulting odds ratios indicated that each 1-year increase in female age reduced euploid odds by 3.5% (OR = 0.965, 95% CI: 0.94–0.99, P = 0.011), and each 5% absolute increment in DFI reduced odds by 9.5% (OR = 0.905, 95% CI: 0.83–0.97, P = 0.006)(**Supplementary Table S5**).

### Subgroup analyses

Based on clinical practice and previous literature, age was categorized into three subgroups: < 35, 35-39, and ≥ 40 years. In the unadjusted analyses, subgroup analyses stratified by female age (**Table 3**; **Figure 4**) revealed that male DFI was associated with lower odds of euploidy in women aged <35 years (nominally significant: OR = 0.87 [0.76-1.00], unadjusted p = 0.046, FDR-adjusted q = 0.166) and 35-39 years (nominally significant: OR = 0.87 [0.75-1.00], p = 0.046, FDR-adjusted q = 0.166), but not in women ≥ 40 years (OR = 0.90 [0.70-1.16], p = 0.425, q = 0.589).Female age showed a nominal association with reduced odds of euploidy only in the 35–39-year subgroup (OR = 0.82 [0.71–0.96]; unadjusted p = 0.011; q = 0.166). Male BMI was associated with reduced odds of euploidy only in the ≥ 40 years subgroup (OR = 0.75 [0.59-0.97], p = 0.026). However, the ≥ 40 subgroup was too small for robust inference. More importantly, in the subgroup analyses stratified by female age, none of the predictors showed a statistically significant association after FDR correction for multiple comparisons (all q > 0.05). All subgroup analyses based on female age strata were conducted as exploratory analyses, requiring validation in future prospective studies.

**Table 3:** Subgroup analysis by female age.

Variable	Age group	number of patients	number of blastocysts	number of euploid blastocysts	OR (95% CI)	unadjusted p-value	FDR-adjusted q-value
Female Age	<35	213	1010	595	1.02 [0.89-1.18]	0.729	0.807
	35-39	273	1034	566	0.82 [0.71-0.96]	0.011*	0.166
	≥40	102	313	138	0.91 [0.72-1.14]	0.408	0.589
Female BMI	<35	213	1010	595	0.98 [0.86-1.11]	0.761	0.807
	35-39	273	1034	566	1.17 [1.01-1.36]	0.032*	0.166
	≥40	102	313	138	1.15 [0.9-1.46]	0.267	0.534
Male Age	<35	213	1010	595	0.92 [0.8-1.06]	0.265	0.534
	35-39	273	1034	566	0.93 [0.8-1.08]	0.322	0.565
	≥40	102	313	138	1.04 [0.81-1.34]	0.762	0.807
Male BMI	<35	213	1010	595	1.1 [0.97-1.25]	0.155	0.465
	35-39	273	1034	566	1.11 [0.95-1.29]	0.205	0.527
	≥40	102	313	138	0.75 [0.59-0.97]	0.026*	0.166
Female AMH	<35	213	1010	595	0.99 [0.87-1.13]	0.867	0.867
	35-39	273	1034	566	1.07 [0.93-1.25]	0.345	0.565
	≥40	102	313	138	0.95 [0.75-1.21]	0.688	0.807
Male DFI	<35	213	1010	595	0.87 [0.76-1]	0.046*	0.166
	35-39	273	1034	566	0.87 [0.75-1]	0.046*	0.166
	≥40	102	313	138	0.9 [0.7-1.16]	0.425	0.589

*p < 0.05 (unadjusted); q-values were calculated using the Benjamini–Hochberg method with a false discovery rate of 0.05. All q-values > 0.05 after FDR adjustment, as shown in the table for each comparison.

**Figure 4:**
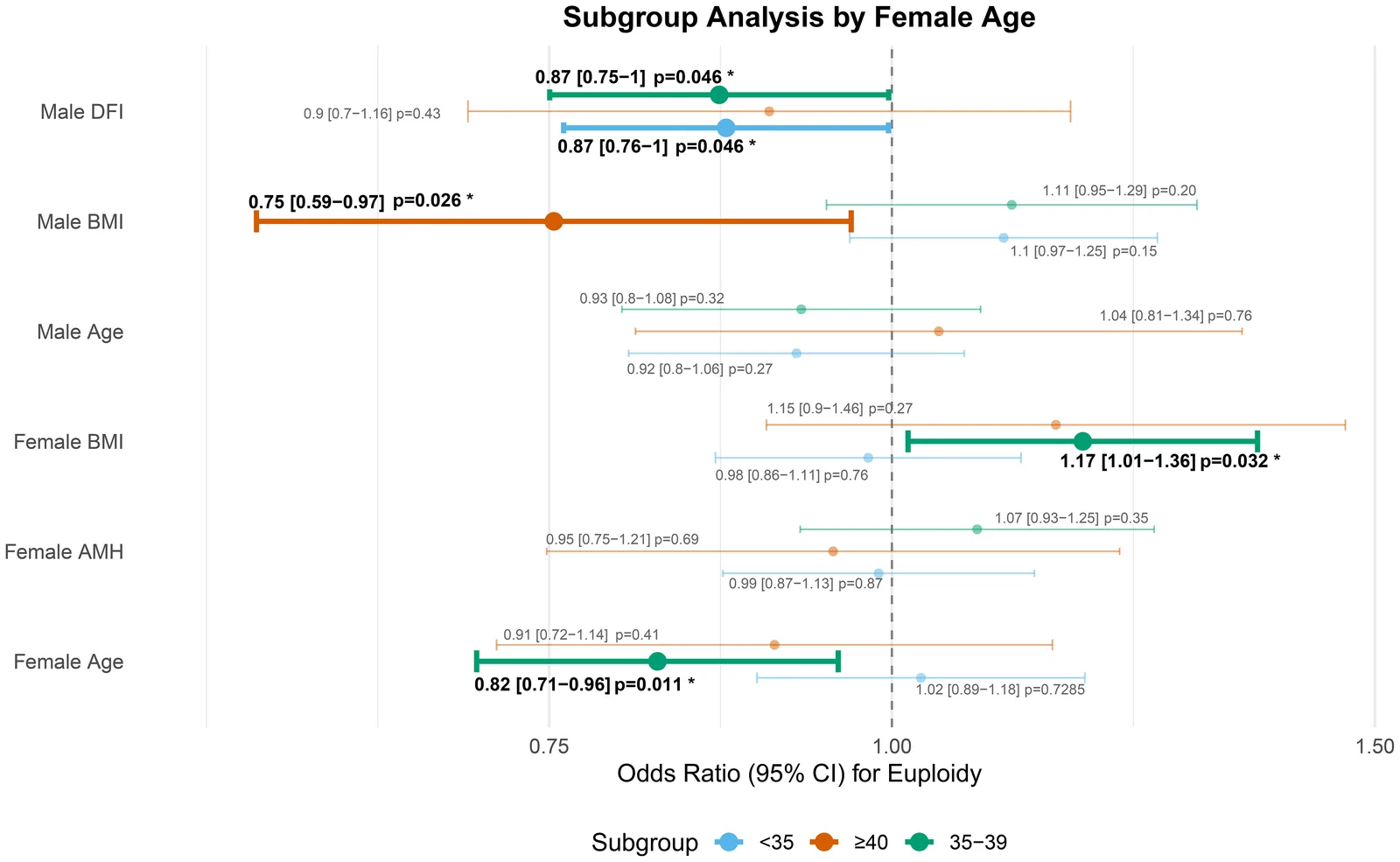
Subgroup analysis forest plot stratified by female age (< 35, 35–39, ≥ 40 years) Cycles were divided into three subgroups based on female age: < 35 years, 35–39 years, and ≥ 40 years. In each subgroup, a multivariable GLMM was used to estimate the odds ratio (OR) and 95% confidence interval (CI) for each factor associated with embryo euploidy rate. Effect estimates are shown for female age, female BMI, male age, male BMI, female AMH, and male DFI. *p < 0.05 (statistically significant).

**Supplementary Table S3** presents subgroup analyses stratified by male DFI. In accordance with the statistical analysis plan described in the Methods, these analyses were conducted as exploratory analyses and are hypothesis-generating. All p-values in **Supplementary Table S3** were adjusted using the Benjamini–Hochberg method, and adjusted q-values are reported. In the ≥ 30% stratum, which comprised only 6 patients (21 embryos), the model did not converge and no odds ratio could be estimated; the corresponding cells are therefore reported as “not estimable” and no inference is drawn from this stratum.

### Interaction analyses

**Supplementary Table S2** showed no significant multiplicative effects between female age and male DFI (OR = 1.03 [0.95-1.12], p = 0.408) or between female BMI and male BMI (OR = 1.04 [0.96-1.12], p = 0.323). As the interaction terms did not reach statistical significance (both p > 0.05), the observed subgroup differences should be interpreted as exploratory observations rather than confirmatory evidence of effect modification.

**Supplementary Table S7** presents the exploratory sequential sensitivity analyses, all of which were fitted on the 1,819-embryo subset (359 patients). Male DFI remained significantly associated with euploidy in all four models (Model 1 OR 0.867, Model 2 OR 0.864, Model 3 OR 0.862, Model 4 OR 0.866). Female age also remained significant throughout, including in Model 4 after adjustment for blastulation day, morphological grade and infertility type (OR = 0.860, 95% CI 0.756–0.977, P = 0.021). The Day 6 association was attenuated after the addition of morphological grade (Model 2: OR = 0.739, 95% CI 0.610–0.895, P = 0.0019; Model 3: OR = 0.847, 95% CI 0.694–1.034, P = 0.103), suggesting that static morphology captures part of the information conveyed by blastulation kinetics; as no formal mediation analysis was performed, this observation should be interpreted descriptively. Morphological grade was the strongest embryo-level correlate (Model 4: OR = 2.147, 95% CI 1.621–2.844, P < 0.001), whereas infertility type (secondary vs. primary) was not associated with euploidy (OR = 1.135, 95% CI 0.894–1.439, P = 0.299). Adding blastulation day alone did not significantly improve discrimination (ΔAUC = +0.008, 95% CI −0.0002 to +0.023), whereas the addition of morphological grade produced a small but consistent improvement (Model 2 → Model 3: ΔAUC = +0.019, 95% CI + 0.009 to +0.034). Overall, however, model performance remained poor (Model 4: apparent AUC = 0.612, marginal R^2^ = 0.052, conditional R^2^ = 0.058, apparent Brier score = 0.238), far below the level required for clinical prediction. To distinguish the effect of sample restriction from that of additional adjustment, Models 1 and 2 were refitted on the full cohort of 2,357 blastocysts (**Supplementary Table S9**). The direction of both parental associations and of the blastulation-day effects was preserved across the two samples. Because the subset was defined by a non-random pattern of missing morphology records, this comparison is presented as a descriptive stability check and is not used to support the primary conclusions.

## Discussion

PGT-A is used to screen the chromosomal composition of embryos generated through assisted reproductive technology (ART) (23, 24). Selection of euploid embryos for transfer minimizes implantation failure and miscarriage risk while improving clinical outcomes. Consequently, euploidy rate is one of the most critical metrics in PGT-A cycles, because it directly influences embryo quality, pregnancy success, and offspring health.

Importantly, our cohort was predominantly composed of RPL patients (70.8%), with a mean female age of 35.8 years; thus, our conclusions are most directly applicable to this specific population, and extrapolation to pure AMA or RIF cohorts requires further validation. The results of the univariate generalized linear mixed-effects model in this study indicated that female age, male age, and male DFI were significantly associated with embryo euploidy (p < 0.001). After multivariate adjustment, advanced female age (OR = 0.87, 95% CI 0.77-0.97, p = 0.011) and male DFI (OR = 0.89, 95% CI 0.81-0.97, p = 0.006) remained independent correlates for embryo euploidy. Female age is widely recognized as the most important risk factor for embryonic aneuploidy (4–6). This association is primarily attributed to the accumulation of meiotic errors, telomere shortening, and mitochondrial dysfunction. In the age-stratified subgroup analysis, the association of female age was nominally significant only in the 35–39-year stratum (OR = 0.82, 95% CI 0.71–0.96; unadjusted p = 0.011; FDR-adjusted q = 0.166), and was not significant in women aged ≥ 40 years (OR = 0.91, 95% CI 0.72–1.14; unadjusted p = 0.408; q = 0.589). Because no stratum-specific association survived FDR correction, these findings are hypothesis-generating rather than evidence of a genuine biological difference between strata. Their estimates should be interpreted cautiously, as statistical power for detecting subtle differences is limited. Larger studies are needed to confirm these findings in this age group.

Male DFI was significantly associated with embryo euploidy in both univariable and multivariable models (p < 0.01). However, this finding contrasts with several previous studies. Yang et al. (25) found no significant difference in aneuploidy rates between high- and normal-DFI groups (44.7% vs 37.8%, P > 0.05); Kong et al. (16) reported no association in 667 blastocysts; and Green et al. (17) likewise found no association between sperm DNA fragmentation on the day of fertilization and embryologic outcomes. Several factors may explain these discrepancies: (i) our cohort was predominantly RPL patients (70.8%), a population differing from unselected IVF/ICSI cohorts; (ii) the DFI assay used here was acridine-orange flow cytometry, whereas other studies used SCSA or TUNEL, with different thresholds and sensitivities; (iii) the DFI distribution was restricted (73.3% <15%; only 1.0% ≥30%), limiting power at the high end; and (iv) DFI was measured once on ICSI day, which may not reflect persistent DFI status. Our findings should therefore be considered exploratory. Elevated sperm DFI may reduce the probability of embryo euploidy by compromising sperm genomic stability and increasing the risk of chromosomal abnormalities. Nominal significant associations between DFI and embryonic euploidy were observed in the < 35 and 35–39 years female age strata in uncorrected subgroup analyses. Nevertheless, no subgroup associations remained significant after Benjamini-Hochberg FDR multiplicity correction, consistent with the non-significant interaction test between female age and male DFI (female age and male DFI, OR = 1.03 [0.95-1.12], p = 0.408). Accordingly, this stratified signal should not be interpreted as evidence of a true effect.

Notably, male/female BMI and female AMH were not independently associated with embryo euploidy in the multivariate model (all p > 0.05), indicating limited predictive value after accounting for confounding variables. After excluding the interference of multicollinearity (all VIFs < 2; **Supplementary Table S6**), AMH remained non-significant in the fully adjusted model (OR = 1.01, 95% CI 0.92-1.10, p = 0.884; **Table 2**). In this cohort, after adjusting for female age and other parental baseline factors, AMH showed no independent association with embryonic euploidy. This supports the view that AMH mainly reflects ovarian reserve (oocyte quantity), though we cannot generalize that AMH is unrelated to oocyte quality across all clinical settings, as its effects may be masked by stronger confounding variables, such as age (26).

Unadjusted subgroup analyses identified several nominally significant associations for female and male BMI; however, none remained significant after FDR correction (all q > 0.05). Given the low statistical power within age strata (EPV range: 23-99), these isolated findings are likely chance observations rather than true interactions, and the interaction analysis was also non-significant. Therefore, these findings should be interpreted with caution and considered exploratory.

The area under the ROC curve (apparent AUC) was only 0.571, suggesting limited discriminative performance for clinical decision‑making. For instance, a study by De Gheselle et al. using morphokinetic features in a random forest model achieved a substantially higher AUC (0.75) than our clinical‑variable GLMM, which showed much lower discriminative ability (corrected AUC = 0.574) (27).

Collectively, these findings indicate that currently available routine clinical variables are far from sufficient for accurately predicting the euploidy status of individual embryos. Embryo euploidy is likely determined to a greater extent by stochastic events, molecular mechanisms during gametogenesis (e.g., meiotic recombination, centromere aging), and genetic factors that are not routinely measured in clinical practice (e.g., mitochondrial DNA copy number, telomere length) (28).

The patient random intercept standard deviation was 0.25(ICC ≈ 0.02), suggesting small between‑patient heterogeneity, yet still supporting the use of mixed models to account for within-patient embryo correlations. Interaction tests did not reveal significant multiplicative interactions between female and male factors (female age × male DFI, p = 0.408), suggesting that the effects of these variables are additive rather than synergistic or antagonistic.

The exploratory sensitivity analyses (**Supplementary Table S7**) were conducted on the subset of 1,819 embryos (359 of 588 cycles) with documented morphological grade, and should therefore be read alongside the primary analysis rather than as confirmation of it. Within this subset, male DFI remained significantly associated with euploidy after sequential adjustment for blastulation day, morphological grade and infertility type, with an effect estimate close to that of the primary model (Model 1 OR 0.867 to Model 4 OR 0.866), and female age likewise remained significant (Model 4 OR 0.860, 95% CI 0.756-0.977, P = 0.021). Because the subset retained 77.2% of embryos but only 61% of cycles and was derived from a non-random pattern of missing records, these estimates are reported as exploratory and are not used to support the primary conclusions. Within this subset, the female-age estimate was of similar magnitude across the sequential models (Model 1 OR 0.834 to Model 4 OR 0.860). Because these four models are nested within the same non-random subset, the stability of the estimate does not establish that the association is independent of unmeasured embryo-level or baseline clinical characteristics, and this cannot be excluded with the present data. Morphological grade substantially attenuated the Day 6 effect, highlighting the overlap of information between static and kinetic embryo parameters; as no formal mediation analysis was conducted, this should be interpreted descriptively. Although adding these embryological parameters — chiefly morphological grade — improved model fit measurably in this subset (apparent AUC from 0.583 to 0.612; marginal R^2^ from 0.024 to 0.052; apparent Brier score from 0.244 to 0.238; **Supplementary Table S7**), discrimination remained far below the threshold required for clinical use, within this exploratory subset, routine clinical and basic embryological variables lack sufficient predictive value. This underscores the need for novel biomarkers and larger cohorts to achieve clinically meaningful predictions. Several limitations should be acknowledged. First, the model explained very little variability (R^2^ < 0.04, AUC = 0.571), indicating that the six factors are insufficient for clinical prediction; unmeasured factors (e.g., aneuploidy origin, follicular microenvironment) likely play larger roles. Second, the small sample size in the ≥ 40 years subgroup widened CIs and reduced statistical power. The age effect was primarily driven by the 35–39 age subgroup and should not be overinterpreted. Third, the retrospective single-center design may introduce bias, and we lacked lifestyle or detailed semen data beyond a single DFI measurement on ICSI day, which may not reflect each patient’s persistent DFI level. Fourth, procedural ART parameters were not incorporated into our primary model; adjustment for these intermediate endpoints could introduce over-adjustment bias and distort effect estimates for parental baseline factors. Moreover, after FDR correction, none remained significant, therefore, the results of our age-stratified subgroup analysis are hypothesis-generating rather than confirmatory, given limited power for interaction detection. The results of internal validation (apparent AUC = 0.571, bootstrap-corrected AUC = 0.574, apparent calibration slope = 0.985, bootstrap-corrected slope = 0.913; **Supplementary Table S4**) indicate only modest optimism in the slope. The over‑representation of RPL cases limits generalizability to RIF or AMA populations, further validation in RIF and pure AMA cohorts is warranted. A further limitation is the restricted DFI distribution in our cohort. Since most participants had normal-range DFI and few had pathological DNA fragmentation, the observed DFI-outcome association cannot be extrapolated to men with clinically elevated DFI. Finally, this study was primarily designed to investigate mechanistic questions with each biopsied blastocyst treated as the analytical unit. Among 588 cycles with complete covariate data, 90.3% had at least one euploid blastocyst; among all 613 biopsied cycles and 637 started cycles, the rates were 89.89% and 86.50%, respectively. This discrepancy should be taken into account when interpreting our findings in the context of clinical counseling and cycle outcome prediction. This conclusion should not be directly extrapolated to all PGT initiation cycles. Clinical decision-making requires careful consideration of other pertinent factors. Larger diverse cohorts integrating embryological covariates and incorporating multi-dimensional biomarkers are needed to confirm our observations. If the 12 excluded mosaics were counted as aneuploid, the euploidy rate would be 54.83% instead of 55.11%, a difference of less than 0.3 percentage points. Our conservative mosaic-reporting policy and the inherent lower sensitivity of targeted NGS for sub-20% mosaicism mean that our 0.5% mosaic rate and 55.1% euploidy rate are not directly comparable to cohorts using a permissive 20–80% reporting rule or whole-genome/SNP-array platforms. Our main findings are restricted to unequivocal euploid and aneuploid embryos because mosaics are biologically ambiguous and not transferred at our center. Thus, extrapolation to mosaics is not warranted; prospective studies with adequate mosaic transfers are needed for full validation. All stratified analyses were exploratory; these exploratory findings require validation in independent cohorts before any clinical translation can be considered. Morphological grade was missing for 538/2,357 blastocysts (22.8%) due to historical database gaps, so fully adjusted and embryo-level sensitivity analyses were restricted to 1,819 blastocysts from 359 patients. Although included and excluded patients were broadly comparable (**Supplementary Table S8**), five of the ten characteristics with a reported SMD showed small but non-negligible imbalance (|SMD| 0.101-0.142) and excluded embryos had a higher euploidy rate (59.7% vs 53.8%). This subset was therefore non-random, and the **Supplementary Table S7** estimates are exploratory and need prospective confirmation. PGT-A indication was not adjusted because its AMA category is defined by maternal age, so residual confounding cannot be excluded, though it is likely limited in a 70.8% RPL cohort.

## Conclusion

Although female age and male DFI showed statistically significant associations with embryo euploidy in the multivariable GLMM, internal validation revealed poor discrimination (apparent AUC = 0.571, bootstrap-corrected AUC = 0.574), whereas calibration remained acceptable after correction (apparent calibration slope = 0.985, bootstrap-corrected slope = 0.913), consistent with weak signal strength rather than miscalibration. These findings indicate that the six routine clinical variables accounted for only a minor fraction of the variability in embryo ploidy, and thus lack sufficient predictive value for reliable clinical application.

On the basis of these results, we caution against using these variables as definitive predictors of embryo euploidy in current practice. Subgroup analyses by female age were exploratory and hypothesis-generating, and no statistically significant age-modified association between male DFI and euploid embryos persisted after FDR correction. Therefore, these variables should not be used alone for prognostic counseling or clinical decision‑making.

Future studies should incorporate a broader range of embryo-specific data and larger sample sizes to develop prediction models for embryo euploidy with greater clinical utility.

## Data Availability

The datasets presented in this article are not readily available because their release is restricted by the Institutional Review Board of the General Hospital of Northern Theater Command. The data that support the findings of this study are available from the corresponding author upon reasonable request. Requests to access the datasets should be directed to YY, yuyuexinpingan@163.com.

## Supplementary material

Supplementary Table 1 Performance metrics of the euploidy prediction model.

Supplementary Table 2 Interaction effects in the multivariable generalized linear mixed-effects model (GLMM).

Supplementary Table 3 Subgroup analysis by male DFI. In the ≥ 30% stratum (n = 6 patients, 21 embryos) the model did not converge and no odds ratio could be estimated; these cells are reported as “not estimable”.

Supplementary Table 4 Internal validation metrics of the multivariable GLMM for predicting embryo euploidy.

Supplementary Table 5 Odds ratios for female age and male DFI scaled to clinically meaningful units.

Supplementary Table 6 Variance inflation factors (VIFs) for the six fixed predictors in the multivariable GLMM.

Supplementary Table 7 Exploratory sequential sensitivity analyses adjusting for embryo‑level characteristics and baseline clinical factors. (N_embryos = 1,819; 77.2% of the primary analysis sample of 2,357).

Supplementary Table 8 Baseline characteristics of cycles included in and excluded from the exploratory sensitivity analysis. (N_cycles = 588; N_embryos = 2,357).

Supplementary Table 9 Replication of the parental-factors model (Model 1) and the blastulation-day model (Model 2) on the full cohort of 2,357 biopsied blastocysts from 588 cycles, with Model 1 additionally refitted on the subset of 1,819 blastocysts from 359 cycles with documented morphology. Model 1 = the six parental factors; Model 2 = Model 1 plus blastulation day. Both are generalized linear mixed models with a patient random intercept (binomial family, logit link; glmer, lme4), as in the primary analysis; continuous predictors were standardized using the SDs of the embryos in the analysis set.
