# Supplementary material for: Parental determinants of blastocyst euploidy in a predominantly recurrent-pregnancy-loss PGT-A cohort: limited predictive value of female age, BMI, AMH and sperm DNA fragmentation
Source: Front Endocrinol (Lausanne). 2026 Oct 28;17:1909930. doi: 10.3389/fendo.2026.1909930 (PMC13751957; doi:10.3389/fendo.2026.1909930)
Supplement: Supplementary file 1 [file Table1.docx]

**Table S1. Performance metrics of the euploidy prediction model.**

| **Metric** | **Value** |
| --- | --- |
| Marginal R² | 0.018 |
| Conditional R² | 0.036 |
| AUC | 0.571 |
| AIC | 3,222.500 |
| BIC | 3,268.600 |

Marginal R², conditional R², AUC, AIC and BIC values for the generalized linear mixed-effects model predicting blastocyst euploidy.
