# Supplementary material for: Parental determinants of blastocyst euploidy in a predominantly recurrent-pregnancy-loss PGT-A cohort: limited predictive value of female age, BMI, AMH and sperm DNA fragmentation
Source: Front Endocrinol (Lausanne). 2026 Oct 28;17:1909930. doi: 10.3389/fendo.2026.1909930 (PMC13751957; doi:10.3389/fendo.2026.1909930)
Supplement: Supplementary file 2 [file Table2.docx]

**Table S2. Interaction effects in the multivariable generalized linear mixed-effects model (GLMM).**

| **Interaction** | **OR** | **CI** | **p-value** |
| --- | --- | --- | --- |
| Female Age × Male DFI | 1.03 | 0.95-1.12 | 0.4082 |
| Female BMI × Male BMI | 1.04 | 0.96-1.12 | 0.3226 |

Two pre‑specified interactions were evaluated: (1) female age × male DFI, and (2) female BMI × male BMI. Odds ratios (OR), 95% confidence intervals (CI), and P-values for interaction terms between parental factors in the adjusted GLMM.
