# Supplementary material for: Parental determinants of blastocyst euploidy in a predominantly recurrent-pregnancy-loss PGT-A cohort: limited predictive value of female age, BMI, AMH and sperm DNA fragmentation
Source: Front Endocrinol (Lausanne). 2026 Oct 28;17:1909930. doi: 10.3389/fendo.2026.1909930 (PMC13751957; doi:10.3389/fendo.2026.1909930)
Supplement: Supplementary file 3 [file Table3.docx]

**Table S3. Subgroup analysis by male DFI.** In the ≥ 30% stratum (n = 6 patients, 21 embryos) the model did not converge and no odds ratio could be estimated; these cells are reported as "not estimable".

| **DFI Subgroup** | **Variable** | **OR (95% CI)** | **p-value** | **FDR‑adjusted q‑value** |
| --- | --- | --- | --- | --- |
| <15% | Female Age | 0.88 [0.77-1.01] | 0.061 | 0.458 |
| <15% | Female BMI | 1.04 [0.94-1.15] | 0.452 | 1.000 |
| <15% | Male Age | 0.92 [0.8-1.05] | 0.202 | 0.758 |
| <15% | Male BMI | 1.04 [0.94-1.16] | 0.423 | 1.000 |
| <15% | Female AMH | 0.99 [0.9-1.1] | 0.912 | 1.000 |
| 15-30% | Female Age | 0.8 [0.65-0.98] | 0.035* | 0.458 |
| 15-30% | Female BMI | 1.18 [0.97-1.43] | 0.1 | 0.5 |
| 15-30% | Male Age | 1.02 [0.83-1.25] | 0.859 | 1.000 |
| 15-30% | Male BMI | 1 [0.84-1.2] | 0.981 | 1.000 |
| 15-30% | Female AMH | 1.06 [0.89-1.27] | 0.498 | 1.000 |
| ≥30% | Female Age | Not estimable |  |  |
| ≥30% | Female BMI | Not estimable |  |  |
| ≥30% | Male Age | Not estimable |  |  |
| ≥30% | Male BMI | Not estimable |  |  |
| ≥30% | Female AMH | Not estimable |  |  |

All analyses are exploratory hypothesis‑generating analyses. Benjamini-Hochberg false-discovery-rate correction was applied across the 10 tests for which a p-value could be estimated (the ≥ 30% stratum did not converge and contributed no p-values). q-values were capped at 1.000. Rows for which the model did not converge are marked "Not estimable" and carry no p- or q-values.
