# Supplementary material for: Parental determinants of blastocyst euploidy in a predominantly recurrent-pregnancy-loss PGT-A cohort: limited predictive value of female age, BMI, AMH and sperm DNA fragmentation
Source: Front Endocrinol (Lausanne). 2026 Oct 28;17:1909930. doi: 10.3389/fendo.2026.1909930 (PMC13751957; doi:10.3389/fendo.2026.1909930)
Supplement: Supplementary file 4 [file Table4.docx]

**Table S4. Internal validation metrics of the multivariable GLMM for predicting embryo euploidy**

| Metric | Value |
| --- | --- |
| Apparent AUC (marginal) | 0.571 |
| Bootstrap-corrected AUC (marginal) | 0.574 |
| Optimism (AUC) | -0.003 |
| Brier score (apparent) | 0.2437 |
| Brier score (bootstrap-corrected) | 0.2433 |
| Apparent calibration slope | 0.985 |
| Slope optimism | 0.072 |
| Corrected calibration slope | 0.913 |
| Apparent calibration intercept | −0.005 |

Abbreviations: AUC, area under the receiver operating characteristic curve; GLMM, generalized linear mixed model.

Note: All metrics are based on marginal predictions (fixed effects only; patient random intercept set to zero) from the final dataset of 2,357 embryos. Bootstrap validation used 200 resamples with patient-level clustering. The apparent calibration slope was computed on the full dataset; optimism was estimated by bootstrap; the corrected slope = apparent slope − optimism. A corrected slope close to 1 and intercept close to 0 indicate good calibration. The low AUC reflects poor discriminative ability, consistent with the low marginal R².A corrected slope below 1 indicates that the apparent predictions are slightly too extreme; here the corrected slope of 0.913 implies shrinkage of approximately 9% on the log-odds scale.
