# Supplementary material for: Parental determinants of blastocyst euploidy in a predominantly recurrent-pregnancy-loss PGT-A cohort: limited predictive value of female age, BMI, AMH and sperm DNA fragmentation
Source: Front Endocrinol (Lausanne). 2026 Oct 28;17:1909930. doi: 10.3389/fendo.2026.1909930 (PMC13751957; doi:10.3389/fendo.2026.1909930)
Supplement: Supplementary file 5 [file Table5.docx]

**Table S5. Odds ratios for female age and male DFI scaled to clinically meaningful units**

| **Predictor** | **OR** | **95% CI** | **p-value*** |
| --- | --- | --- | --- |
| Female age (per +1 year) | 0.965 | 0.94–0.99 | 0.011 |
| Male DFI (per +5% absolute) | 0.905 | 0.83–0.97 | 0.006 |

Continuous variables were standardized (mean = 0, SD = 1) prior to modeling. The odds ratios (ORs) presented above were obtained by rescaling the standardized regression coefficients using the original standard deviations of each variable.

Estimates were derived from the standardized multivariate GLMM coefficients using the actual standard deviations of the predictors in the study cohort SD (female age) = 3.95 years; SD(DFI) = 5.81%.

*p-values are the same as the corresponding standardized estimates.
