# Supplementary material for: Parental determinants of blastocyst euploidy in a predominantly recurrent-pregnancy-loss PGT-A cohort: limited predictive value of female age, BMI, AMH and sperm DNA fragmentation
Source: Front Endocrinol (Lausanne). 2026 Oct 28;17:1909930. doi: 10.3389/fendo.2026.1909930 (PMC13751957; doi:10.3389/fendo.2026.1909930)
Supplement: Supplementary file 6 [file Table6.docx]

**Table S6. Variance inflation factors (VIFs) for the six fixed predictors in the multivariable GLMM**

| Predictor | VIF |
| --- | --- |
| Female age | 1.68 |
| Female BMI | 1.05 |
| Male age | 1.69 |
| Male BMI | 1.08 |
| Female AMH | 1.08 |
| Male DFI | 1.04 |

All VIF values were < 2 (maximum = 1.69), well below the conventional threshold of 5. This indicates that there is no strong correlation among female age, male age, BMI, AMH, and DFI, and each variable contributes independent predictive information to the model.
