# Supplementary material for: Parental determinants of blastocyst euploidy in a predominantly recurrent-pregnancy-loss PGT-A cohort: limited predictive value of female age, BMI, AMH and sperm DNA fragmentation
Source: Front Endocrinol (Lausanne). 2026 Oct 28;17:1909930. doi: 10.3389/fendo.2026.1909930 (PMC13751957; doi:10.3389/fendo.2026.1909930)
Supplement: Supplementary file 7 [file Table7.docx]

**Table S7. Exploratory sequential sensitivity analyses adjusting for embryo-level characteristics and baseline clinical factors.(N_embryos = 1,819; 77.2% of the primary analysis sample of 2,357)**

| **Model** | **Predictor** | **OR (95% CI)** | **p value** | **n (high / low quality)** | **N_embryos** | **N_patients** |
| --- | --- | --- | --- | --- | --- | --- |
| Model 1: Parental factors only | Female Age | 0.834 (0.737–0.943) | 0.0038 | — | 1,819 | 359 |
|  | Male DFI | 0.867 (0.788–0.954) | 0.0036 | — | 1,819 | 359 |
| Model 2: + Day | Female Age | 0.841 (0.742–0.952) | 0.0062 | — | 1,819 | 359 |
|  | Male DFI | 0.864 (0.784–0.951) | 0.0029 | — | 1,819 | 359 |
|  | Day 6 | 0.739 (0.610–0.895) | 0.0019 | — | 1,819 | 359 |
|  | Day 7 | 0.712 (0.322–1.574) | 0.402 | — | 1,819 | 359 |
| Model 3: + Day + Quality | Female Age | 0.851 (0.750–0.966) | 0.0128 | — | 1,819 | 359 |
|  | Male DFI | 0.862 (0.781–0.951) | 0.0031 | — | 1,819 | 359 |
|  | Day 6 | 0.847 (0.694–1.034) | 0.103 | — | 1,819 | 359 |
|  | Day 7 | 0.926 (0.411–2.087) | 0.853 | — | 1,819 | 359 |
|  | High quality ( vs. low quality) | 2.134 (1.610–2.826) | <0.001 | 1,536 / 283 | 1,819 | 359 |
| Model 4: + Day + Quality + Type | Female Age | 0.860 (0.756–0.977) | 0.021 | — | 1,819 | 359 |
|  | Male DFI | 0.866 (0.785–0.956) | 0.004 | — | 1,819 | 359 |
|  | Day 6 | 0.853 (0.698–1.043) | 0.121 | — | 1,819 | 359 |
|  | Day 7 | 0.935 (0.415–2.107) | 0.871 | — | 1,819 | 359 |
|  | High quality ( vs. low quality) | 2.147 (1.621–2.844) | <0.001 | 1,536 / 283 | 1,819 | 359 |
|  | Infertility type: secondary vs. primary | 1.135 (0.894–1.439) | 0.299 | — | 1,819 | 359 |

Models are GLMMs with patient random intercept. All continuous predictors were standardized.

Model 1 included all six pre-treatment parental factors (female age, female BMI, AMH, male age, male BMI and male DFI), of which only the two statistically significant terms are shown; Models 2–4 add embryo-level terms sequentially.

Quality: 1 = high quality (ICM and TE both graded A or B); 0 = low quality (either ICM or TE graded C). Denominators: high quality n = 1,536 embryos, 876 euploid (57.03%); low quality n = 283 embryos, 102 euploid (36.04%). Type: infertility type  (primary as reference vs. secondary). CC-grade blastocysts were excluded from all analysis datasets; therefore, all 283 low-quality embryos were derived from biopsied blastocysts with at least one C grade (but not double C).

 PGT-A indication (RPL / RIF / AMA) was not included as a covariate, because its AMA category is defined by maternal age (≥ 37 years) and is therefore collinear with female age.

Analyses restricted to embryos with documented blastocyst morphological grade; blastulation day was available for all 2,357 embryos. Exclusions due to missing morphological grade records (n = 538). All models: n_embryos = 1,819, n_patients = 359.

| Metric | Model 1 | Model 2 | Model 3 | Model 4 |
| --- | --- | --- | --- | --- |
| Apparent AUC | 0.583 | NR | NR | 0.612 |
| ΔAUC vs previous model (95% CI) | — | +0.008 (−0.0002 to +0.023) | +0.019 (+0.009 to +0.034) | — |
| Marginal R² | 0.024 | NR | NR | 0.052 |
| Conditional R² | NR | NR | NR | 0.058 |
| Brier score (apparent) | 0.244 | NR | NR | 0.238 |

Performance metrics are computed on marginal predictions (fixed effects only, patient random intercept set to zero), as specified in the Methods (lines 233-234 and 241-242), and are apparent values. Internal validation with optimism correction was pre-specified for the primary analysis only (Methods, lines 237-243); the sequential models reported here are exploratory (Methods, lines 261-278) and optimism-corrected metrics were not pre-specified for them, so they are not reported. For Models 2 and 3, incremental discrimination was pre-specified to be reported as ΔAUC with 95% confidence intervals obtained from 200 patient-clustered bootstrap replicates using paired resampling (Results, lines 397-399); absolute values for these two intermediate models were not part of the pre-specified output and are marked NR. The conditional R² of Model 1 is likewise NR for the same reason. The summed ΔAUC estimates (+0.008 + 0.019 = +0.027) differ from the difference between the endpoint apparent AUCs (0.612 - 0.583 = +0.029) by 0.002, which lies within the Monte Carlo error of the bootstrap procedure stated in the manuscript (0.002-0.004). All metrics are computed on the 1,819-embryo subset and are not directly comparable with the primary full-cohort estimates in Table S4, which are based on a different sample.
