# Supplementary material for: Parental determinants of blastocyst euploidy in a predominantly recurrent-pregnancy-loss PGT-A cohort: limited predictive value of female age, BMI, AMH and sperm DNA fragmentation
Source: Front Endocrinol (Lausanne). 2026 Oct 28;17:1909930. doi: 10.3389/fendo.2026.1909930 (PMC13751957; doi:10.3389/fendo.2026.1909930)
Supplement: Supplementary file 8 [file Table8.docx]

### **Table** S8. Baseline characteristics of cycles included in and excluded from the exploratory sensitivity analysis. (N_cycles = 588; N_embryos = 2,357)

| **Characteristic** | **Included (n = 359)** | **Excluded (n = 229)*** | **Full cohort (n = 588)** | **SMD** |
| --- | --- | --- | --- | --- |
| Female age (years) | 35.55 ± 3.92 | 36.09 ± 3.98 | 35.76 ± 3.95 | −0.138 |
| Female BMI (kg/m²) | 23.69 ± 3.60 | 23.67 ± 3.43 | 23.68 ± 3.53 | +0.005 |
| Female AMH (ng/mL) | 3.37 ± 3.11 | 3.02 ± 2.55 | 3.23 ± 2.91 | +0.121 |
| Male age (years) | 36.75 ± 5.09 | 37.14 ± 5.52 | 36.90 ± 5.26 | −0.075 |
| Male BMI (kg/m²) | 25.89 ± 3.70 | 25.71 ± 3.96 | 25.82 ± 3.80 | +0.050 |
| Male DFI (%) | 12.40 ± 5.88 | 12.99 ± 5.69 | 12.63 ± 5.81 | −0.102 |
| RPL, n (%) | 260 (72.42) | 156 (68.13) | 416 (70.75) | +0.094 |
| RIF, n (%) | 51 (14.21) | 41 (17.89) | 92 (15.64) | −0.100 |
| AMA, n (%) | 48 (13.37) | 32 (13.99) | 80 (13.61) | −0.018 |
| Secondary infertility, n (%) | 272 (75.77) | 159 (69.43) | 431 (73.30) | +0.142 |
| Embryos per patient | 5.07 | — | 4.01 | — |
| Euploidy rate (embryo level) | 53.77% (978/1,819) | 59.67% (321/538) | 55.11% (1,299/2,357) | — |

Values for excluded cycles were derived by subtracting the included subgroup from the full cohort. Means and SDs are algebraically consistent with Table 1 (verified).

SMD compares the included with the excluded group: for continuous variables, the difference in means divided by the pooled SD; for binary variables, the difference in proportions divided by the pooled SD of the two proportions.

Applying this criterion, five of the ten characteristics with a reported SMD exceed the 0.10 threshold and therefore show small but non-negligible imbalance: secondary infertility +0.142, female age −0.138, female AMH +0.121, male DFI −0.102, and RIF −0.100 (exact value −0.1009). All five remain below 0.15 and are therefore small in magnitude, but none is negligible under the 0.10 criterion stated above.
