# Supplementary material for: Parental determinants of blastocyst euploidy in a predominantly recurrent-pregnancy-loss PGT-A cohort: limited predictive value of female age, BMI, AMH and sperm DNA fragmentation
Source: Front Endocrinol (Lausanne). 2026 Oct 28;17:1909930. doi: 10.3389/fendo.2026.1909930 (PMC13751957; doi:10.3389/fendo.2026.1909930)
Supplement: Supplementary file 9 [file Table9.docx]

**Table S9. Replication of the parental-factors model (Model 1) and the blastulation-day model (Model 2) on the full cohort of 2,357 biopsied blastocysts from 588 cycles, with Model 1 additionally refitted on the subset of 1,819 blastocysts from 359 cycles with documented morphology. Model 1 = the six parental factors; Model 2 = Model 1 plus blastulation day. Both are generalized linear mixed models with a patient random intercept (binomial family, logit link; glmer, lme4), as in the primary analysis; continuous predictors were standardised using the SDs of the embryos in the analysis set.**

| **Model** | **Predictor** | **OR (95% CI)** | **p value** | **N embryos** | **N cycles** |
| --- | --- | --- | --- | --- | --- |
| Model 1: Parental factors only | Female age | 0.865 (0.774–0.967) | 0.011 | 2,357 | 588 |
|  | Male DFI | 0.886 (0.812–0.966) | 0.006 | 2,357 | 588 |
| Model 1: refitted on the subset with documented morphology | Female age | 0.837 (0.742–0.944) | 0.004 | 1819 | 359 |
|  | Male DFI | 0.869 (0.791–0.955) | 0.004 | 1819 | 359 |
| Model 2: + Blastulation day | Female age | 0.874 (0.788–0.970) | 0.012 | 2,357 | 588 |
|  | Male DFI | 0.874 (0.800–0.956) | 0.003 | 2,357 | 588 |
|  | Day 6 (vs Day 5) | 0.702 (0.579–0.852) | 0.0003 | 2,357 | 588 |
|  | Day 7 (vs Day 5) | 0.661 (0.314–1.393) | 0.276 | 2,357 | 588 |

Model 1 is the six-predictor model of Table 2 (female age, female BMI, female AMH, male age, male BMI, male DFI); only the two statistically significant terms are shown. Model 2 adds blastulation day (reference: Day 5). Odds ratios are per 1 SD; the standardising SDs were those of the 2,357 embryos in the analysis set (female age 3.95 years; male DFI 5.48 percentage points). All odds ratios, 95% CIs and P values were exported from the fitted model object at three-decimal precision and were not transcribed from another table; CIs and P values are Wald-type. N = 2,357 blastocysts from 588 oocyte-retrieval cycles; 12 mosaic embryos were excluded from the 2,369 biopsied blastocysts. The subset rows (1,819 blastocysts from 359 cycles) reproduce Model 1 of Table S7, re-expressed on the same (full-cohort) SD scale; they are shown solely to separate the effect of sample restriction from that of additional adjustment, are descriptive, and are not used to support the primary conclusions.
